# Measurement of Horse Allergen (Equ cx) in Schools

**DOI:** 10.5402/2011/574258

**Published:** 2012-01-04

**Authors:** Anne-Sophie Merritt, Gunnel Emenius, Lena Elfman, Greta Smedje

**Affiliations:** ^1^Institute of Environmental Medicine, Karolinska Institutet, SE-171 77 Stockholm, Sweden; ^2^Department of Public Health Sciences, Karolinska Institutet, SE-171 76 Stockholm, Sweden; ^3^Department of Medical Sciences, Occupational and Environmental Medicine, Uppsala University, SE-751 85 Uppsala, Sweden

## Abstract

*Background*. The presence of horse allergen in public places is not well-known, unlike for instance cat and dog allergens, which have been studied extensively. The aim was to investigate the presence of horse allergen in schools and to what extent the influence of number of children with regular horse contact have on indoor allergen levels. *Methods*. Petri dishes were used to collect airborne dust samples during one week in classrooms. In some cases, vacuumed dust samples were also collected. All samples were extracted, frozen and analysed for Equ cx content shortly after sampling, and some were re-analysed six years later with a more sensitive ELISA assay. *Results*. Horse allergen levels were significantly higher in classrooms, in which many children had horse contact, regardless of sampling method. Allergen levels in extracts from Petri dish samples, which had been kept frozen, dropped about 53% over a six-year period. *Conclusion*. Horse allergen was present in classrooms and levels were higher in classrooms where many children had regular horse contact in their leisure time. This suggests that transfer of allergens takes place via contaminated clothing. Measures should be taken to minimize possible transfer and deposition of allergens in pet-free environments, such as schools.

## 1. Introduction

In Sweden, horseback riding has become increasingly popular and is today practiced by 6% of the population, of which the majority are living in densely built-up areas. A recent survey by the Swedish Board of Agriculture estimated the number of horses to be approximately 363.000 in 2010, an increase of 10–20% since 2004 [1]. Most of the horses (75%) are located in city areas and adjoining rural areas and as a consequence, more stables are integrated with built-up areas. In an attempt to protect susceptible subjects from undesirable exposure to horse allergen, a Swedish report from the Ministry of Health and Social Affairs, in 1989, recommended a distance of at least 500 m between public buildings and homes and places where horses are kept on a regular basis [2]. This recommendation has raised a debate around the issue of whether the level of exposure to horse allergen in the surroundings of stables is different compared to exposure occurring elsewhere, for instance in public environments such as schools. In order to investigate the airborne horse allergen dispersal, a few studies have been performed using different sampling techniques to assess horse allergen levels at various distances from stables and horse tracks [3–5]. The results show that horse allergen levels are elevated in the immediate vicinity of stables and horse fields or other places where horses are present. However, horse allergen levels drop quickly with increasing distance from the source [3, 5].

We and others have previously shown that furred pet allergens are present in schools and that the allergens are readily dispersed and transferred between different environments, mainly via clothing [6–10]. It has been shown that a higher rate of cat ownership among children in a classroom is correlated with increased levels of cat allergen [6, 11, 12] and the same is valid for dog ownership and dog allergen levels [13]. The presence of furred pet allergens in “pet-free” public environments, such as schools, may exacerbate symptoms in allergic children sensitized to furred animals, particularly children with asthma [14–17]. Since school attendance is mandatory, providing a good environment for all children is an obligation according to the Swedish law [18]. Most previous studies have focused on the presence of cat or dog allergen, and only a few have investigated the presence and implication of horse allergen exposure at school [13, 19]. Furthermore, in most of these studies, pet allergens were analysed in dust collected by vacuuming floors and other surfaces and not in airborne dust samples. Samples of airborne allergen should give a better reflection of the actual personal exposure. In previous studies, we and others have used Petri dishes for passive sampling of allergens in settled dust, a method which has proven to be simple and reliable [12, 20]. However, horse allergens may be less abundant compared to other allergens in public environments and hence, a validation of Petri dish sampling in relation to other sampling techniques, such as vacuuming, is therefore desirable.

The aims of the present study were to investigate the presence of horse allergen in schools and to study correlation between allergen levels in schools and number of children with regular horse contact in their leisure time. For this purpose, air and dust samples from three different school exposure studies have been analysed for the content of the horse allergen Equ cx. To our knowledge, no such large-scale assessment of horse allergen levels in schools has been compiled before.

## 2. Materials and Methods

### 2.1. Study Design and Allergen Sampling

Air and dust samples from three different school exposure studies were analysed for the content of horse allergen Equ cx.

*Study I*: a single, coated Petri dish (base and lid) was used for sampling during one school week in approximately 200 primary and secondary schools in the county of Stockholm (totally, 293 classrooms), at inner-city, suburban, and rural locations. The sampling procedure has been described and validated in previous studies [12, 20]. Briefly, a teleostean gelatine-coated Petri dish (*∅*15 cm) was placed with base and lid facing upwards at 150 cm above floor level on a desk or equivalent. The Petri dishes were placed in classrooms during one school week from Monday morning to Friday afternoon. Samples were analysed with the enhanced horse allergen ELISA in 2005 (see below).

*Study II:* a single noncoated Petri dish (base and lid) was placed in the classroom during one school week. Measurements were performed in 116 classrooms from 35 primary and secondary schools in the county of Uppsala north of Stockholm. The schools were situated in both inner-city and rural locations. Sampling of vacuumed dust from floors and furniture was also performed, directly after collecting the Petri dishes as described previously [11, 17]. Briefly, a dust collector (ALK Abello, Copenhagen, Denmark) attached to a regular vacuum cleaner was used. In all classrooms, two samples were taken, each by vacuuming half the classroom during 4 minutes, equally divided between floor and furniture. For each classroom, the mean concentration from the two samples was calculated and expressed as U/g dust.

Both Petri dish samples and vacuumed samples were analysed in 2003 with the standard horse allergen ELISA assay. Petri dish samples were analysed again in 2009 (*n* = 97), using the enhanced horse allergen ELISA assay, which had been developed since the first time of analysis.

*Study III:* during a 10-week period, weekly measurements were carried out in 20 classrooms in four primary schools in a suburban municipality of Stockholm. Coated Petri dishes were placed at 150 cm above floor level on aluminium holders attached to the wall. Samples were analysed with the enhanced horse allergen ELISA method.

In all three studies, information on the number of children, who had regular contact with horses and other furry animals was gathered. Information about distances between schools and horse stables was obtained from the local municipality councils.

### 2.2. Allergen Extractions

All Petri dishes were kept at room temperature until extraction (on average 2 weeks). Allergen extraction of airborne, settled dust collected with Petri dishes was performed as follows. In Study I, Petri dishes were extracted in 3 mL phosphate-buffered saline with 0.05% Tween 20 (PBS-T) and the extract was then transferred to a tube without subsequent rotation and centrifugation before storage [12]. In Study III, Petri dishes were extracted with 2 mL PBS-T. The extract was then transferred to a tube and rotated overnight at room temperature, and finally centrifuged at 1000x*g* for 10 min [8]. In study II, 3 mL PBS-T containing 1% BSA was added to the base and incubated during gentle shaking for one hour at room temperature after which the extract was transferred to the lid and extraction continued for another hour. The extract was then transferred to an Eppendorf tube and centrifuged at 7000x*g* for 10 minutes [19]. Vacuumed dust samples were weighed and 100 mg of dust was extracted in 2 mL PBS-T as described earlier [13]. All extracted samples were kept at −20°C in aliquots until allergen analysis.

### 2.3. ELISA Analysis of Horse Allergen, Equ cx

Vacuumed dust samples and Petri dish samples from Study II were analysed for horse allergen, Equ cx, with a monoclonal sandwich ELISA (from now on referred to as the “standard ELISA assay”), used and described previously using reagents from Mabtech (Nacka, Sweden) [3, 4]. This ELISA assay was developed further and all Petri dish samples were reanalysed several years later using this more sensitive assay (“enhanced ELISA assay”), as described below.

Microtiter plates (Maxisorp, Nunc, Roskilde, Denmark) were coated over night at 4–8°C with the anti-horse mAb 103, at 2 *μ*g/mL in 50 mM carbonate/bicarbonate buffer (pH 9.6). After washing, plates were blocked for 60 minutes. Standards (range 0.125 to 8 U Equ cx/mL, similar to ng Equ cx/mL [3]), samples and controls were added in duplicate and incubated for 90 minutes. Biotinylated monoclonal antibody 14G4, diluted 1/2000, was added and after 60 minutes of incubation, streptavidine polyhorseradish peroxidase (Mabtech, Nacka, Sweden), diluted 1/1000, was added and plates were incubated for 60 minutes. Finally, plates were developed with 100 *μ*L K-Blue (Neogen Corp., ANL-Produkter, Älvsjö, Sweden) and the colour reaction was stopped by adding 100 *μ*L 1 M H_2_SO_4_. Absorbance was measured at 450 nm using a plate reader and concentration levels were interpolated using a 4-parameter curve fit. The detection limit of this enhanced, more sensitive ELISA assay is 0.2 U/mL, compared to 2 U/mL with the standard ELISA assay.

Horse allergen analyses with the enhanced ELISA assay were performed six years after sampling. The horse allergen used as a standard was a kind gift from Phadia AB (Uppsala, Sweden). The horse allergen extract was developed as described earlier [3].

Samples from Study II, initially analysed in 2003 using the standard ELISA assay, were compared with results obtained with the new, enhanced ELISA to estimate the effect of the storage time between sampling and analysis (2003, *n* = 116 and 2009, *n* = 97).

Since both coated (Study I and III) and uncoated (Study II) petri dishes were used, a small validation study was performed to investigate the importance of using teleostean gelatine coating [12, 20] by parallel sampling with coated and uncoated Petri dishes. No differences were found, hence uncoated dishes were used in Study II.

### 2.4. Statistical Calculations

For statistical calculations, samples below the detection limit in the enhanced ELISA assay (0.2 U/mL) were assigned a value of 0.1 U/mL and likewise, 1 U/mL for the standard ELISA assay. Allergen levels were expressed as units (U) Equ cx/m^2^/day (Petri dishes) or U/g dust (vacuumed dust). “Day” was adjusted according to number of school days with children present, which was generally 5 days. In Study III, the mean value of two Petri dish samples was used per sampling week and classroom. To estimate reduction of allergen content due to storage in Study II, only samples which were above the detection limit in the 2003 assay were used. The geometric mean of the allergen reductions (percentage) was calculated. Correlations were calculated using Spearman's rank correlation. Comparisons were made using the unpaired Mann-Whitney *U*-test.

## 3. Results

**Figure 1:**
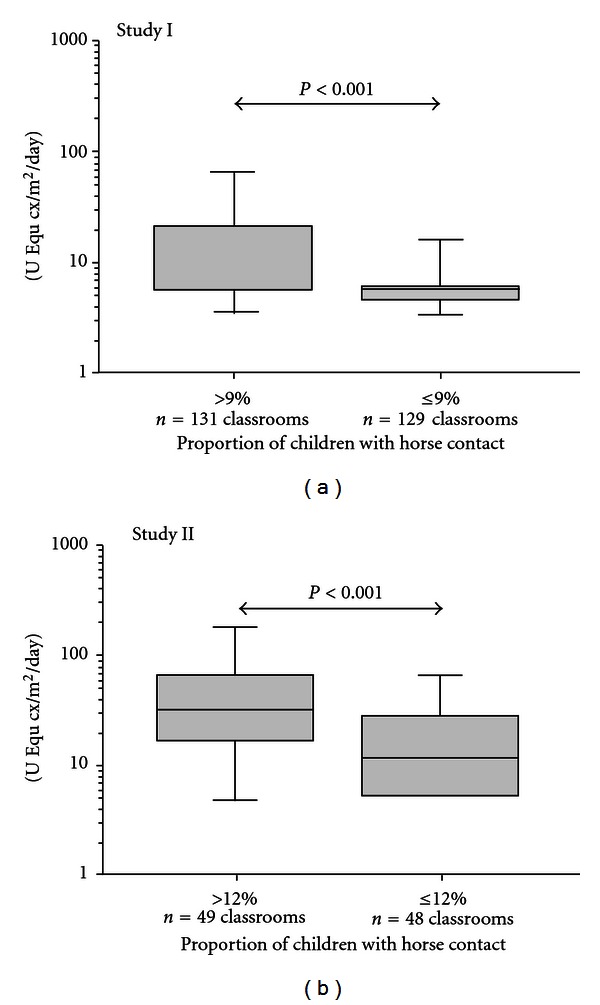
Levels of horse allergen in airborne settled dust collected with Petri dishes in classrooms (Study I and II).

Results from Study I and II showing the levels of horse allergen are presented in Table 1. Many samples were below the detection limit (0.2 U/mL or 2 U/mL for the standard ELISA assay) in the ELISA assays but horse allergen could still be detected in many classrooms (37%). Overall, about one in ten of the school children reported having regular horse contact (9, 12, and 8% for Study I, II, and III, resp.). In classrooms, where the percentage of children with horse contact was above the median rate of reported horse contact, the levels of horse allergen sampled with Petri dishes were significantly higher, compared to those in classrooms where children reported less or no horse contact (below median rate of reported horse contact, Figure 1). In Study I, we did not get information about horse contact among children in 33/293 (11%) classrooms; hence samples collected from those classrooms were not included in the calculations regarding influence of rate of horse contact.

**Figure 2:**
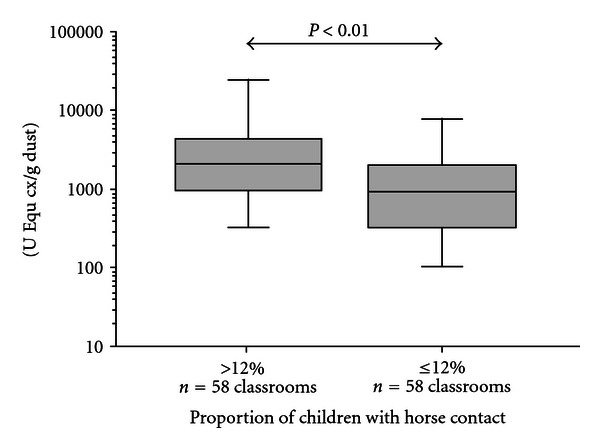
Levels of horse allergen in vacuumed dust samples in classrooms (Study II).

**Figure 3:**
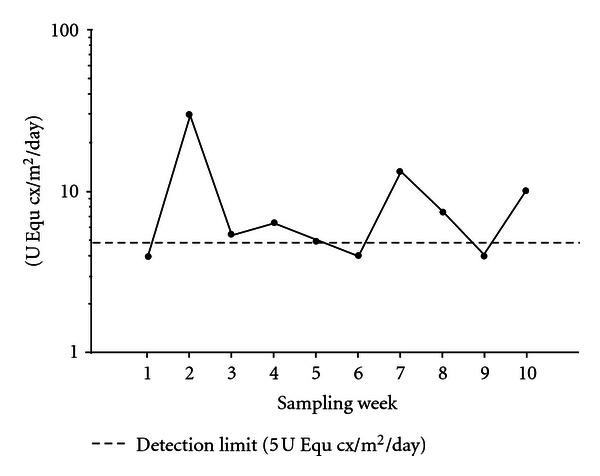
Levels of horse allergen in airborne settled dust collected with Petri dishes in classrooms (*n* = 20) every week for 10 weeks (Study III).

A positive relationship between horse allergen levels and rate of horse contact was also shown for vacuumed dust samples in Study II (Figure 2).  Figure 3 illustrates how allergen levels vary between sampling weeks during a 10-week study period in 20 classrooms (Study III). The dotted line represents the approximate detection limit for the allergen levels expressed as 5 U/m^2^/day.

In Study II, in which extracted Petri dish samples were analysed promptly after sampling (2003) as well as six years later (2009), the allergen content in the frozen, extracted samples had dropped on average by about 53% (18–98%).

The correlation between the two analyses (2003 and 2009) was 0.73. For this calculation, only samples which were above the detection limit in both assays were included. The rationale behind this was that the ELISA-assay used in 2009 was more sensitive with a lower detection limit of 0.2 U/mL instead of 2 U/mL.

Both sampling methods, Petri dishes and vacuumed dust, were used in 116 classrooms, and samples were analysed with the standard ELISA assay in 2003. The geometric mean horse allergen concentrations are shown in Table 2. In classrooms where many children reported horse contact (>12%), the levels of horse allergen were significantly higher (for both sampling methods), compared to levels obtained in classrooms where children reported less or no horse contact (<12%). For Petri dish samples, 72% of the samples were below the detection limit, but for vacuumed samples only 4%. The correlation between the two methods was 0.32, with a *P* value below 0.000.

## 4. Discussion

In the present study, horse allergen, Equ cx, was detectable both in airborne and settled dust collected from schools and levels were influenced by the number of children who reported regular horse contact in their leisure time. Most schools were situated far away (>500 m) from stables which shows that close vicinity to horses is not a requirement for obtaining detectable levels of horse allergen indoors.

Clothing which have been used in stables are generally not used or even permitted inside school premises in Sweden, but contamination of clothing may occur, for instance when traveling by public transport. Horse allergens are indeed present in buses, at underground stations, and grocery/food stores (manuscript in preparation). In addition, other routes of exposure, such as via hair, may also influence the levels of horse allergen in schools, as has been shown for cat allergen [21].

In Study III, we could show that levels of horse allergen, both between and within classrooms, fluctuated between weeks. A classroom with 20–30 students present, is a dynamic environment and variable allergen levels can be expected as a result of movements [8]. Therefore, it is important to consider repeated measurements when assessing allergen exposure in indoor environments.

The extended storage time (six years) of extracted and frozen samples prior to allergen analysis with the enhanced ELISA assay resulted in reduced horse allergen concentrations, as shown in Study II. Hence, an overall underestimation of airborne horse allergen exposure is most likely the case in our study. We found that horse allergen levels in extracted and frozen (−20°C) dust samples were more than halved over a six-year-storage period. During development of the standard horse allergen ELISA assay, a validation test of the horse allergen standard was performed, using both stressed and real-time studies. The horse allergen was shown to be stable for at least 2 years at 4–8°C, but less stable at frozen state [3]. Fahlbusch et al. looked at house dust samples and found that storage at −20°C for up to 10 months had no effect on mite allergen, while Fel d 1 concentrations significantly declined with increased storage time [22].

Allergen levels were considerably higher in Study II and fewer samples (28/97 or 29%) were below the detection limit (<0.2 U/mL) compared with 74% in Study I. The most feasible explanation may be that BSA was added to the extraction buffer in Study II, which is known to stabilize the allergen content during storage and perhaps most importantly, to reduce unspecific binding to plastic vials. In addition, the extraction procedure used in Study II may be more efficient in extracting the allergens from the dust particles, although this has not been evaluated. However, the percentage of pupils with regular horse contact was higher in Study II than in Study I and III, this is why the observed difference in allergen levels may reflect a true difference.

In this study, we mainly used Petri dishes for allergen sampling, a method which is easy and cheap to perform, and collects particles that have been airborne. Although the sampling method has been evaluated for cat allergen previously [12], the question is whether it is the most appropriate sampling method for assessment of horse allergen exposure. However, for analysis of airborne cat allergen in our previous studies, an amplified ELISA assay was used, which is more sensitive (detection limit 2.5 pg/mL) than the enhanced horse allergen ELISA (detection limit 0.2 U/mL = approximately 0.2 ng/mL). As a consequence, a substantial proportion of the Petri dish samples were below the detection limit. The vacuumed dust sampling method detected horse allergen in more classrooms than the airborne Petri dish sampling method. Both vacuumed dust samples and Petri dish samples showed significant relationships to the rate of children with horse contact, which strengthens the validity of both methods. However, the correlation between Petri dish and vacuumed dust samples was rather low (rho = 0.32). Similar results have previously been found for dog (Tau-beta = 0.5, *P* < 0.001) and cat (Tau-beta = 0.31, *P* = 0.032) allergen in air and dust samples [13]. Vacuumed dust from surfaces, floors, and textiles are commonly used in exposure assessments, but should be regarded only as a proxy for personal exposure. Measurement of airborne allergens by using personal breathing zone samplers may be a more accurate method for assessment of personal exposure [23].

Another important issue is whether this indirect exposure to horse is clinically relevant for sensitive individuals or may even induce sensitisation, as one study suggests [24]. A previous study from Uppsala found that current asthma and respiratory symptoms were more prevalent among students attending a school with high levels of horse allergen in vacuumed dust, compared to schools with lower levels [17]. Previously, a relationship between asthma and cat allergen at school has been found. Almqvist et al. showed that cat-sensitized children with asthma had more symptoms when returning to school after summer holidays, if they attended a class with many cat owners [15] and Smedje and Norbäck found that the incidence of asthma was higher among pupils attending schools with more cat allergen [16]. To our knowledge, the study by Kim and coworkers is the only published study investigating health effects of horse allergen exposure at school. The impact of this indirect exposure to horse at school on symptoms still remains to be clarified. A study by Novembre et al. showed that very few children were sensitized [25] and/or had symptoms to horse, hence the exposure-symptom relationship is difficult to evaluate.

However, Liccardi et al. reported that allergic sensitization to horse allergen is more common (3.4%) than expected in subjects living in urban areas and without direct exposure to horses [24].

Still, it seems reasonable to suggest that allergen exposure should be kept at a minimum in the school environment. A common practise should be to always change clothing and shower before leaving the stables. However, many people travel to and from stables dressed in horse clothing, often on public transport where allergens are deposited. Furthermore, many stables do not provide changing room facilities with showers, which is common in most other sport facilities.

In conclusion, horse allergen levels are present in classrooms, although there are no stables nearby. Allergen levels, found both in airborne and vacuumed dust, are higher in classrooms where many children have regular horse contact in their leisure time, hence measures should be taken to minimize the transferal and deposition of allergens in such pet-free environments.

Whether the above described passive exposure levels to horse allergen causes any adverse health effects in sensitive individuals remains to be elucidated.

## Figures and Tables

**Table 1:** Horse allergen levels in classrooms from Study I and II, sampled with Petri dishes.

	Study I	Study II
Sampling year	1997/1998	2003
Analysis year (enhanced ELISA assay)	2005	2009
Median horse contact among students	9%	12%
No. of samples	293	97
Samples < dl	74%	29%
GM U/m^2^/day (Total)	8.4	20.8
GM U/m^2^/day (in classes above median rate of horse contact)	10.5	32.6
GM U/m^2^/day (in classes below median rate of horse contact)	6.3	13.2

**Table 2:** Horse allergen levels in vacuumed dust and airborne settled dust from Petri dishes in Study II.

	Vacuumed dust (U/g dust)	Petri dish (U/m^2^/day)
Sampling year	2003	2003
Analysis year (standard ELISA assay)	2003	2003
No. of samples	116	116
Samples < dl	4%	72%
GM (total)	1343	73.9
GM, classes >12% horse contact	2051	96.2
GM, classes ≤12% horse contact	880	56.7
